# Relationships between Demographic Factors and Chronic Conditions with Disease Severities

**DOI:** 10.3390/ijerph182111469

**Published:** 2021-10-31

**Authors:** Van Cuong Nguyen, Jungmin Park

**Affiliations:** School of Nursing, Hanyang University, Seoul 04763, Korea; cuongnv@hanyang.ac.kr

**Keywords:** chronic disease, demographic, disease, mortality, severity of illness, prevalence

## Abstract

Disease severities are the outcomes of an inpatient visit classification that assigns a diagnostic related group, including risk of mortality and severity of illness. Although widely used in healthcare, the analysis of factors affecting disease severities has not been adequately studied. In this study, we analyze the relationships between demographics and chronic conditions and specify their influence on disease severities. Descriptive statistics are used to investigate the relationships and the prevalence of chronic conditions. To evaluate the influence of demographic factors and chronic conditions on disease severities, several multinomial logistic regression models are performed and prediction models for disease severities are conducted based on National Inpatient Sample data for 2016 provided by the Healthcare Cost and Utilization Project database in the United States. The rate of patients with a chronic illness is 88.9% and the rate of patients with more than two chronic conditions is 67.6%; further, the rate is 62.7% for females, 73.9% for males, and 90% for the elderly. A high level of disease severity commonly appears in patients with more than two chronic conditions, especially in the elderly. For patients without chronic conditions, disease severities show a lower or safe level, even in the elderly.

## 1. Introduction

Chronic conditions (CC) are a topic of much interest because of their influence and importance in the healthcare and treatment of patients in recent years. Multiple chronic conditions, referred to as comorbidity, are used to refer to patients who have at least two chronic conditions at the same time [1]. This concept has been quite widely used by healthcare professionals in clinical practice and health policy documents [2,3,4,5,6,7]. Multimorbidity has been used to refer to patients who have at least three chronic conditions at the same time [8]. The criterion of three chronic conditions has been considered to be a more valid cut-off in elderly patients treated in the ambulatory care setting, instead of the usual criterion of two chronic conditions [8,9]. Patients with many chronic conditions often have certain difficulties and place a burden on health facilities, and so are associated with high healthcare costs because having more than one disease requires complex disease management, including treatment and self-care [10,11,12,13,14,15,16,17]. In this study, the concept of chronic conditions is spontaneously used to refer to the number of chronic conditions suffered by a patient. This is done to avoid confusion in implementation, moreover, the differences between patients without chronic conditions, patients with only one or two chronic conditions, and patients with three or more chronic conditions are clearly specified.

Disease severity measures, including the risk of mortality and severity of illness, are the outcomes of an inpatient visit classification system that assigns a diagnostic related group [18]. They are widely used to characterize the impact of a disease process on the utilization of resources, comorbidities, and mortality [18,19,20]. Thus, disease severity status has a high impact on mortality rates [18]. The early determination of a disease severity level helps many health facilities simultaneously examine and make the best treatment plan for patients, something that is especially important for patients with three or more chronic conditions [18,21].

Based on previous studies, the prevalence of people with multiple chronic conditions ranges from 16% to 58% in UK studies, 26% in US studies, and 9.4% in urban South Asian studies [2]. As the average span of a person’s life increases, the number of people with many chronic conditions increases significantly [14,16,17,22]. The prevalence of chronic conditions in patients changes also by demographic factors [13,17,23]. For instance, the proportion of black people with three or more chronic conditions is much higher than white people [23]. Healthcare providers have struggled to manage chronic conditions as they bring adverse effects such as severe disease, which is highly positively correlated with disability rates and mortality rate [13]. Thus, it is necessary to classify and identify the characteristics of patients with each chronic condition in their epidemiological relationships with demographic factors, including age, sex, and race, and analyze the impact of these factors on patients’ disease severities. However, previous studies lack specific information about the relationship between demographic factors, chronic conditions, and patients’ disease severities [18,19,21].

The purpose of this study is to identify if there is a relationship between demographics and chronic conditions, and to provide quantitative analysis to specify the influence of demographic factors as well as chronic conditions on patients’ disease severities, include estimates and predictions of disease severities which may help in the provision of healthcare.

## 2. Materials and Methods

### 2.1. Data and Variable Definitions

The inpatient data from the National Inpatient Sample for 2016 (NIS 2016 data) are used, which were provided by the Healthcare Cost and Utilization Project (HCUP) database of the United States, including nine months of medical records (from 1 January 2015, to 30 September 2015). The NIS 2016 data contain information from all patients whether they were insured or uninsured. The NIS 2016 sampling frame comprises 46 states and the District of Columbia, covering more than 97% of the United States population and including almost 96% of discharges from United States community hospitals.

Demographic factors used in this study included age, sex, and race. For research purposes, only patients aged 18 years or older were used, and to indicate differences by age, the AGE variable in the NIS 2016 data was separated into four age groups: early working age (18–24 years), prime working age (25–54 years), mature working age (55–64 years), and elderly (65 years or older), based on the United States age structure [24]. The RACE variable divided the patients into six racial and ethnic groups: White, Black, Hispanic, Asian and Pacific Islander, Native American, and Other [25]. The risk of mortality (ROM) and severity of illness (SOI) are two measures of disease severities [18]. Both ROM and SOI in the NIS 2016 data were divided into five levels corresponding to disease severity levels, numbered 0 through 4. Level 0 was considered the lowest, while level 4 referred to extreme severity level [25].

During this study, we used the chronic disease classification method introduced in previous literature [8] and ICD 10-CM [26] to code diseases. Specifically, chronic diseases were classified into 46 major disease groups called “chronic conditions” (CC). The results of previous studies showed that the list covered all chronic diseases with prevalence rates of at least 1% in elderly patients [8,10,11]. As an example of the chronic conditions, the chronic diseases with ICD-10-CM codes F00-03, F05.1, G30, G31, and R54 were grouped under the “dementia” chronic condition. To avoid repetition, a complete list of 46 chronic conditions was not included. A patient was considered chronically ill only if at least one of the conditions on the list is present [8]. The CC variable was determined based upon the number of chronic conditions found in the patient and was divided into four categories, corresponding to patients without chronic conditions, with only one chronic condition, with two chronic conditions, and with more than two chronic conditions. Categories of these variables are described in detail in the following Table 1.

### 2.2. Methods

This study provides several descriptive statistics to show the prevalence of CC and the differences in demographic characteristics between a patient without chronic conditions, with a single chronic condition, with two chronic conditions, or a patient with more than two chronic conditions. The differences in the expression of patients’ disease severities for each demographic factor and CC are also indicated. The Chi-square test [27] is used to calculate *p* values for the differences across a patient’s sex, CC categories, or disease severity levels. The obtained results are shown in Section 3.1, Section 3.2 and Section 3.3.

Multinomial logistic regression models [28] are performed to evaluate the influence of demographics and CC on patient’s disease severities. To conduct the models, the dependent variables are disease severities (ROM and SOI), and the independent variables are demographic factors (AGE, SEX, and RACE) and CC. There are two multinomial logistic models that correspond with two dependent variables ROM and SOI. They are defined as:

(1) $$p_{i}{}^{\left(R\right)}=P\left(ROM=i\right),\;\;p_{i}{}^{\left(S\right)}=P\left(SOI=i\right),\;i=0,\;1,\;2,\;3,\;4$$

The values of $p_{i}{}^{\left(R\right)}$, $p_{i}{}^{\left(S\right)}$ show the corresponding probabilities for a patient who has the ROM and the SOI at i-th level, i=0, 1, 2, 3, 4.

The reference categories are specified to conduct the multinomial logistic regression models for both models. Here, the category corresponding to level 0, considered the safest level, is used to designate the reference. Subsequently, the other categories are separately regressed against the reference. The general multinomial logistic regression models are shown in the following equations.

(2) $$\begin{array}{ccc} {\mathrm{logit}}_{i}^{\left(R\right)}=\log\left(\frac{p_{i}^{\left(R\right)}}{p_{0}^{\left(R\right)}}\right) & = & \beta_{i}^{\left(0\right)}+\sum_{j=1}^{3}\beta_{ij}^{\left(age\right)}(AGE=j)+\beta_{i}^{\left(sex\right)}(SEX=1)\\ & + & \sum_{j=2}^{6}\beta_{ij}^{\left(race\right)}(RACE=j)+\sum_{j=1}^{3}\beta_{ij}^{\left(cc\right)}(CC=j) \end{array}$$

and

(3) $$\begin{array}{ccc} {\mathrm{logit}}_{i}^{\left(S\right)}=\log\left(\frac{p_{i}^{\left(S\right)}}{p_{0}^{\left(S\right)}}\right) & = & \alpha_{i}^{\left(0\right)}+\sum_{j=1}^{3}\alpha_{ij}^{\left(age\right)}(AGE=j)+\alpha_{i}^{\left(sex\right)}(SEX=1)\\ & + & \sum_{j=2}^{6}\alpha_{ij}^{\left(race\right)}(RACE=j)+\sum_{j=1}^{3}\alpha_{ij}^{\left(cc\right)}(CC=1) \end{array}$$

The regression coefficients are typically jointly estimated by maximum a posteriori (MAP), an extension of the maximum likelihood method. The Wald test [27] is used to determine the statistical significance of estimates. The goodness of fit test [29] is used to test the suitability of models.

The coefficient represents the change in the log-odds ratio (or the relative risk ratio) of the dependent variable’s difference in a particular category compared with the reference category, associated with a one-level change of the respective independent variable.

The following formulas are used to obtain the predicted probabilities for each level of disease severity.

(4) $$p_{i}^{\left(R\right)}=p_{0}^{\left(R\right)}*\exp\left({\mathrm{logit}}_{i}^{\left(R\right)}\right),p_{i}^{\left(S\right)}=p_{0}^{\left(S\right)}*\exp\left({\mathrm{logit}}_{i}^{\left(S\right)}\right),i=1,2,3,4$$

where

(5) $$p_{0}^{\left(R\right)}=\frac{1}{1+\sum_{i=1}^{4}\exp\left({\mathrm{logit}}_{i}^{\left(R\right)}\right)}\;\mathrm{and}\;p_{0}^{\left(S\right)}=\frac{1}{1+\sum_{i=1}^{4}\exp\left({\mathrm{logit}}_{i}^{\left(S\right)}\right)}$$

For the results fitted by the models, we report the estimates of the regression coefficients and the corresponding odds ratios with a 5% level of significance. For the purpose of prediction, all achievable possibilities are considered and calculated for each case, then an average with a standard error is reported. The obtained results are shown in Section 3.4,.

## 3. Results

### 3.1. Characteristics of Patients

The characteristics of patients are described in Table 2. There is a total of 893,967 patients, of which females comprise 506,189 (56.62%), while males comprise only 387,778 of the patients (43.38%). By AGE, the elderly (coded by 3) have the highest percentage with 44.22% (395,274 patients). Among them, females account for a higher percentage than males with 54.66% (216,047 patients). Patients of prime working age (coded by 1) rank second with 300,732 patients (33.64%), of which females also account for a higher percentage than males with 62.57% (188,179 patients). Ranked third is patients of mature working age (coded by 2) with 17.16% (153,446 patients), however, in this group, the proportion of males (53.78%) is higher than that of females (46.22%). Patients of early working age account for the lowest percentage in this study (4.98%), of which females account for more than twice as many males (69.74% vs. 30.26%). Regarding RACE, white patients (coded by 1) account for the highest percentage with 69.79% (623,895 patients), of which females account for 55.53%, higher than males (44.47%). Followed by black patients (coded by 2) with 13.53% (120,984 people), of which females continue to account for a higher percentage than males with 58.10%. Hispanic patients (coded by 3) are ranked third with 9.19% (82,115 people), and females account for a higher percentage than males with 60.21%. The remaining racial groups (coded by 4–6) account for less or less significant numbers, with proportions of 4.83%, 0.16%, and 2.5%, respectively. Finally, in terms of CC, patients with at least one chronic condition account for a very high rate with 88.89%, including 10.63% with only one chronic condition (coded by 1), 10.71% with 2 chronic conditions (coded by 2), and 67.55% of patients with three or more chronic conditions (coded by 3). A rather interesting finding here is that although the proportion of females is consistently higher than that of male patients, while the proportion of males tends to increase with the number of chronic conditions, there is a tendency of decreasing trend in females. For patients without chronic conditions, females account for a much higher percentage than males (78.79% vs. 21.21%).

### 3.2. Prevalence of Chronic Conditions

The distribution of CC by demographic factors is shown in Table 3. By AGE, the proportion of patients with 3 or more chronic conditions in the elderly is very high (over 90%) and then it gradually decreases in the younger age groups. Specifically, in patients of mature working age, it is over 78%, it is 40.57% for prime working age, and nearly 14% for early working age. Meanwhile, the proportion of patients without chronic conditions in early working age (41.5%) is much higher than in the other age groups, even the proportions of mature working age and the elderly are very small (2.74% and 0.88%, respectively). The proportion of patients with only one chronic condition or two chronic conditions continue to decline with increasing age, however, these proportions of mature working age and the elderly increase significantly compared with patients without chronic conditions. By SEX, the proportion of patients with three or more chronic conditions is 73.87% in males, higher than in females (with 62.71%). However, the proportion of patients without chronic conditions is much higher in females than in males (15.46% vs. 5.43%). Regarding RACE, the proportion of patients without chronic conditions among Asians and Islanders is 29.65%, higher than all those of other racial groups. The proportion of patients with three or more chronic conditions in this group is also the lowest (44.95%). Meanwhile, for white patients, the proportion of patients without chronic conditions is only 8.73%, but it is very high for patients with three or more chronic conditions (71.91%). For black patients or native Americans, the proportion of patients with three or more chronic conditions accounts for about 64%.

**Figure 1:**
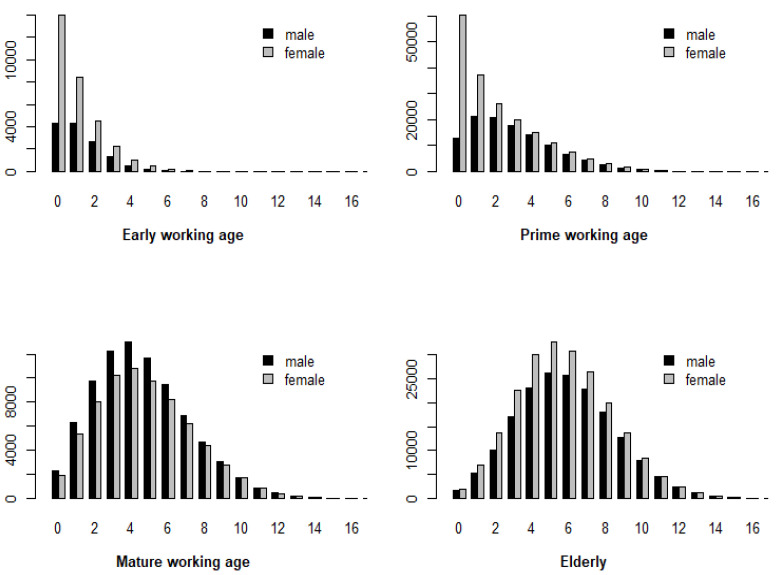
Prevalence of chronic conditions (CC) in patients. The horizontal axis shows the number of chronic conditions, and the vertical axis shows the corresponding counts.

The prevalence of CC in patients is shown in detail in Figure 1. With regard to the visual aspect, there is a difference between male and female patients here. For patients of mature working age, the number of females corresponding to each CC is always less than that of males. Conversely, in the other ages, females corresponding to each CC outperform males.

### 3.3. Disease Severity Measures

Disease severity measures, including ROM and SOI, are essential factors in the prognosis and treatment of disease. As shown in Table 4, consider the ROM aspect, of the total amount of patients, those at level 1 account for the highest rate with 48.71%, followed by patients at level 2 with over 25%. Rates for patients at higher ROM levels are 19.15% at level 3 and 7.1% at level 4 while the rate of patients at level 0 is negligible (only 0.03%). By AGE, patients of early working age have the highest percentage at level 1 with over 88%, followed by level 2 with 7.45% and the lowest at level 0 with a nominal proportion of 0.05%. The ROM varies in descending order at level 1 and in ascending at more dangerous levels (2–4) as age increases, while the ROM at level 0 is negligible. This makes ROM in the elderly open to the highest percentage at level 2 with over 34% and level 3 with over 32%. The extreme level (level 4) of the ROM for the elderly has increased significantly by 11.48%. In comparison, level 1 drops to 22.29%. Concerning SEX, there is a similarity in the order of the ROM in levels for both males and females (the highest rates are at level 1, followed by level 2, level 3, and the lowest at level 0). Nonetheless, there is a slight difference in the proportions of patients at each level; specifically, these rates on females correspond to levels 1–4, and 0, respectively, with 54.04%, 22.84%, 17.17%, 5.93% and 0.02% and in males with 41.75%, 27.85%, 21.75%, 8.61%, and 0.03%, respectively. In terms of RACE, although there is no change in the order of the ROM in levels for six racial groups, coded 1 to 6 (the highest is still level 1, followed by levels 2–4, and 0), there is a clear difference in the ROM levels for the groups. White patients have the most force among racial groups, however, their ROM at level 1 has the lowest rate (45.42%); nevertheless, at higher levels (2–4), this racial group has the highest rate (with 26.03%, 20.77%, 7.75%, respectively). For patients who have less than three chronic conditions, the ROM at level 1 particularly predominates, it is 93.82% for patients without chronic conditions, 81.71% for those with only one chronic condition, and 68.53% for those with two chronic conditions. However, the rates of patients at higher ROM levels increase rapidly as the CC increases. Specifically, for patients without chronic conditions, the ROM at level 2 is only 3.67%, it is 1.44% at level 3, and is even less than 1% at level 4. On the other hand, for patients with three or more chronic conditions, the ROM at level 2 is 31.82%, approximately 26% at level 3, and at the highest ROM level is 9.25%. This shows the extent of the danger posed by CC.

Consider the SOI aspect shown in Table 4, of the total number of patients, those at level 2 account for the highest rate with 40.24%, followed by those at level 1 with 26.24% and those at level 3 with 25.92%. The rate for patients at the highest ROM level is 7.57% while the rate of patients at level 0 is negligible (only 0.03%). For early working-age patients, the SOI at level 1 is the highest with over 47%, followed by level 2 with around 40% and level 3 with 10.4%. Similar to the ROM aspect, the rates of patients at high levels (3 and 4) increase rapidly as age increases. In terms of SEX, the SOI at level 2 is the highest and is similar for both males and females (about 40%); however, at higher levels (3 and 4), these rates are higher for males than females. In terms of race, SOI at level 2 is the highest for all racial groups. White people have an SOI at levels 3 and 4 with a slightly higher incidence than the rest of the racial groups. The Hispanic and Asian and Pacific Islanders groups have an SOI at level 1 with higher rates than the rest. SOI at level 1 predominates with over 60% for patients without chronic conditions, followed by level 2 with 32.45%, while SOI at level 4 is only 1.12%. The rates of patients at high SOI levels (3 and 4) also increasing rapidly as CC increases. Specifically, for patients with only one chronic condition, SOI at level 3 is 11.37%, and it is 3.18% for SOI at level 4. These rates become 16.24% and 5.27%, respectively for patients with two chronic conditions and they increase extremely for patients with three or more chronic conditions (32.98% and 9.68%, respectively).

**Figure A1:**
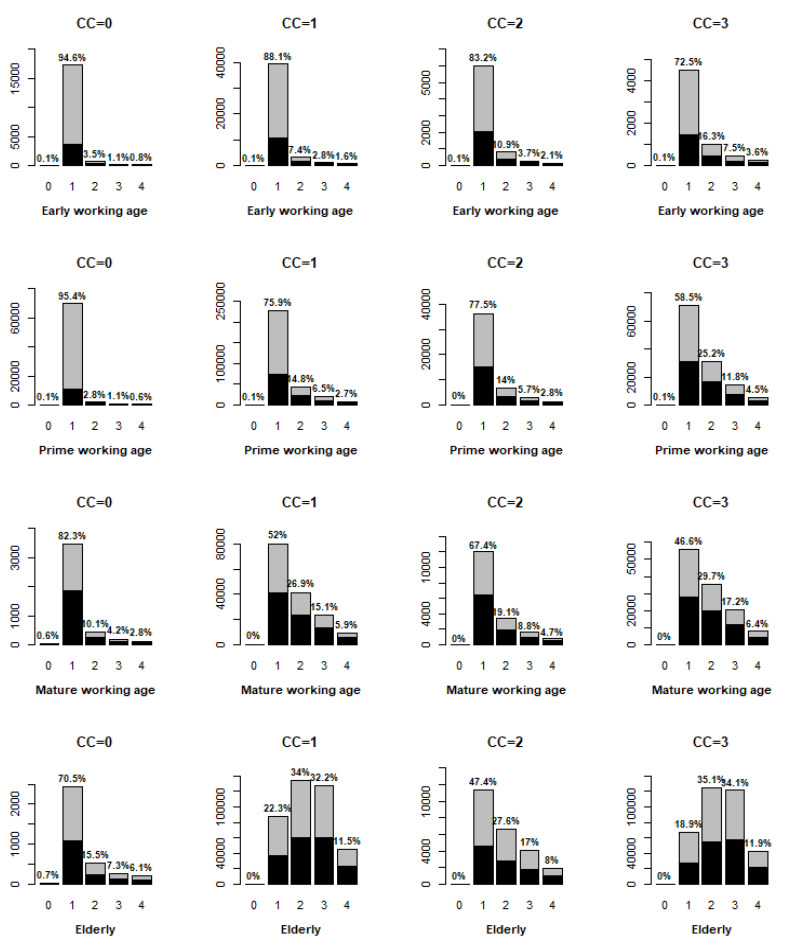
ROM and differences in patients’ characteristics among demographic factors and CC. The black is for male and grey is for female.

**Figure A2:**
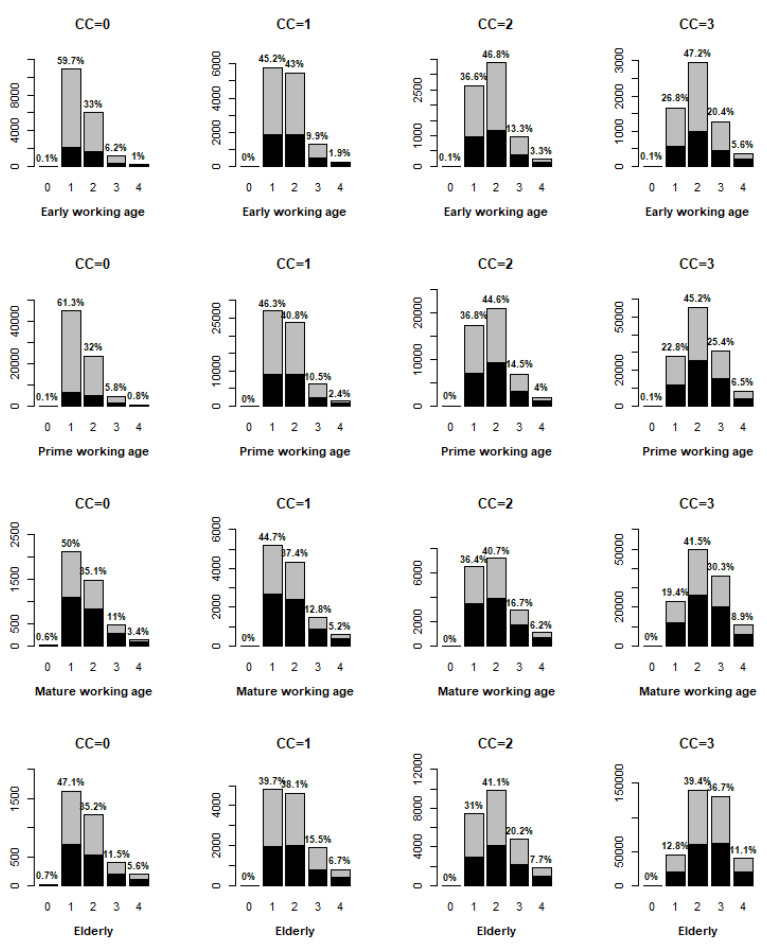
SOI and differences in patients’ characteristics among demographic factors and CC. The black is for male and grey is for female.

The disease severities of patients vary according to demographic factors and CC. The older the patient, the more severe patient’s disease severities are, especially for patients with many chronic conditions. Figure A1 and Figure A2 describe the differences in patient’s disease severities among demographic factors and CC.

### 3.4. Multinomial Logistic Regression Analysis

The results of multinomial logistic regression analyses are shown in Table 5. For a 5% level of confidence, all of the attributes are statistically significant. The extremely small *p*-value in the goodness of fit test (around 2.2 × 10^−16^) means that the models are appropriate and consistent. In other words, the demographic factors and CC indeed affect disease severities. Odds ratios, which are obtained by exponentiation of the regression coefficients, show the association as well as the influence of factors with disease severities. An odds ratio of 1 means there is no influence while the further away the odds ratio from 1 is, the stronger is the influence [21]. As shown in Table 5, for both ROM and SOI, elderly patients (AGE = 3) are strongly correlated with disease severities, particularly with high ROM and SOI levels. Sex is also a very important factor in determining disease severities. By racial groups, Hispanics have the strongest influence. There is an especially powerful influence of the CC factor on disease severities.

The biggest advantage of using multinomial logistic regression models is to provide predictive results. This tells us what kind of level of disease severity a patient with given demographic characteristics and CC is likely carrying and what achievable probabilities they can expect. This can then act as a guide for healthcare facilities in disease control and in developing treatment plans for patients. For example, consider a patient with the demographic characteristics of being elderly, male, Hispanic, and without chronic conditions (AGE = 3, SEX = 0, RACE = 3, and CC = 0), the predicted probability for ROM is 0.13% at level 0; 69.62% at level 1; 14.67% at level 2; 9.69% at level 3; and 5.89% at level 4. For SOI, the predicted probabilities for the patient to fall into the 0–4 levels are 0.09%; 45.19%; 38.42%; 13.25%; and 3.05%, respectively. The age factor has influenced the disease severities, although there are no chronic conditions. The following examples show the dangers of CC. Consider a patient described as elderly, female, Asian and Pacific Islanders race, and having more than two chronic conditions (AGE = 3, SEX =1, RACE = 4, CC = 3). The predicted probability for this patient to fall into ROM is 0% at level 0; 22.09% at level 1; 33.90% at level 2; 32.52% at level 3; and finally 11.49% at level 4. For SOI, the predicted probabilities for this patient to fall into the 0–4 levels are 0%; 14.95%; 39.81%; 34.37%; and 10.87%, respectively. It is clear for this patient that both ROM and SOI are of great concern. One more example, consider the patient characterized by (AGE = 0, SEX = 1, RACE = 1, and CC = 3). The predicted probabilities for this patient are as follows. ROM is 0.01% at level 0; 70.61% at level 1; 17.70% at level 2; 8.82% at level 3; and 3.45% at level 4. ROM at level 1 is the highest but is also of concern at higher levels. Further, SOI is 0.01% at level 0; 24.67% at level 1; 48.56% at level 2; 22.19% at level 3; and 4.57% at level 4. The predicted probability of falling into level 2 is the highest, followed by level 1, level 3, and level 4.

Nevertheless, the patient’s information is not always complete; some factors of demographics and CC may be lacking. In such cases, we calculate and give an average with a standard error based on all achievable possibilities that the patient will likely encounter the respective levels of disease severities. For example, to provide a predictive result for a female patient without chronic conditions, lacking the information of age and race, all potential outcomes of AGE or RACE are considered, with only one average value reported later. To highlight the powerful influence of the CC factor on disease severities, the predictive results for patients with only one demographic factor are presented and compared with patients with more information regarding their CC. The predictive accuracy is reflected through the standard error criterion. For example, if the patient is of mature working age (AGE = 2) with three chronic conditions (CC = 3) (sex and race factors are unknown), the predicted probabilities are as follows: ROM is in 45.35% at level 1; 30.68% at level 2; 17.42% at level 3; and 6.54% at level 4. The corresponding standard errors are 5.09%; 2.24%; 2.03%; and 1.15%. Similarly, SOI is 19.07% at level 1; 41.69% at level 2; 30.24% at level 3; and 8.99% at level 4 with corresponding standard errors 2.71%; 1.11%; 2.33%; and 1.54%. The detailed results are shown in Table A1.

## 4. Discussion

Disease severity measures, including risk of mortality (ROM) and severity of illness (SOI), are the criteria used to describe the impact of the disease process on the utilization of resources, comorbidities, and mortality [18,19,20]. Early identification of disease severity helps health facilities in the provision of the best treatment plan for patients [21], which is especially important for patients with multiple chronic conditions. This study shows the relationships between demographic factors, including AGE, SEX, RACE, and chronic conditions (CC) and their influence on patient’s disease severities, based on NIS 2016 data. The differences between a patient without chronic conditions; with a single chronic condition; with two chronic conditions or a patient with more than two chronic conditions are specified. Simultaneously, the prevalence of CC and the differences in expression of patient’s disease severities for each demographic factor and CC are also indicated. This study uses the NIS 2016 data.

ROM and SOI are often closely related, but the level of ROM and SOI is not always the same for each patient. As shown in Table 6, among patients with an SOI level of 1, just 91.40% of those have a ROM level of 1; among patients with an SOI level of 2, only 37.35% of those have a ROM level of 2, while 55.28% of patients have a ROM level of 1, and so on. The percentage of patients with ROM levels corresponding to each SOI level is indicated in the following matrix. This explains the simultaneous study of both ROM and SOI instead of focusing on only one aspect.

This study uses a natural classification of CC, including patients without chronic conditions, with a single chronic condition, with two chronic conditions, or patients with three or more chronic conditions. We find that the proportion of patients with three or more chronic conditions in diagnosed patients is over 67%. It is over 62% for only female patients and approximately 74% for male patients. For elderly patients, it is over 90% while only around 14% for patients of early working age. This is different but not contradictory compared with previous studies because the prevalence of chronic conditions is calculated based only on inpatients instead of the whole US population [3,6]. The proportion of patients with more than two chronic conditions increases with age. This proportion also varies with the patient’s race. White people have the highest proportion (nearly 72%), the Asians and Pacific Islanders have the lowest proportion (nearly 45%). This is consistent with previous studies [13,23].

The obtained results show that a patient’s disease severities vary with demographic and CC factors. As age increases, the proportions of patients with low levels (including levels 0 and 1) decrease while increasing rapidly in higher levels of disease severities (including grades 2–4). Especially in the elderly, disease severities are at very high levels. High levels of disease severity account for greater proportions of patients with three or more chronic conditions, while for patients without chronic conditions these proportions are very small and insignificant. These results are new and have not been found in detail in previous studies. The results obtained by the multinomial logistic regression analysis are considered a quantification of the influence of demographic and CC factors on disease severities. The older the patient is, the more pronounced the disease severities are. If the relative risk ratio of patients in primary working age compared with early working age according to ROM at levels 2–4 only increases by 0.95, 0.98, and 0.83 times respectively, this rate increases by 1.77, 2.15, and 1.83 times respectively for patients in mature working age. For elderly patients, this rate increased by 5.91, 12.03, and 9.44 times, respectively. In particular, the relative risk ratio of patients with three or more chronic conditions compared with those without chronic conditions according to ROM at levels 2–4 increases as high as 39.93, 54.87, and 31.25 times, respectively. This is similar to SOI.

Furthermore, the increasing trend of CC, as well as relative risk ratios for elderly patients, may significantly increase disease severities. Here, we provide prediction models through multinomial logistic regression models even if patient information is lacking or incomplete. The predictive results show that the models are appropriate and consistent with NIS 2016 data. This contributes to the provision of more quality information about diseases, helping healthcare facilities make appropriate plans for diagnosing and treating diseases for patients.

As a limitation, this study did not include all demographic factors such as income, location, and hospital. However, this study is useful to describe the influence of demographic factors on disease severities. The results of the study demonstrate the relationship between disease severities, demographic factors (including age, sex, race) and CC. For future studies, other demographic factors as well as lifestyle factors will be included based on the methods presented in this study.

## 5. Conclusions

This study shows that there is a significant relationship between demographic factors, chronic conditions and a patient’s disease severity. The differences between patients corresponding to each chronic condition level in the levels of disease severities are revealed. A high level of disease severity commonly appears in patients with more than two chronic conditions, especially in the elderly. For patients without chronic conditions, disease severities are revealed to be at a lower or safe level, even in the elderly.

## Figures and Tables

**Table 1:** Description of variables.

Variable	Category	Description
AGE (Age in years)	0	Early working age, patient aged 18 to 24 years
	1	Prime working age, patient aged 25 to 54 years
	2	Mature working age, patient aged 55 to 64 years
	3	Elderly, patient aged 65 years or older
SE x(Patient’s sex)	0	Male
	1	Female
RACE (Patient’s race)	1	White
	2	Black
	3	Hispanic
	4	Asian and Pacific Islander
	5	Native American
	6	Other
ROM (Risk of mortality)	0	No class specified
	1	Minor likelihood of dying
	2	Moderate likelihood of dying
	3	Major likelihood of dying
	4	Extreme likelihood of dying
SOI (Severity of illness)	0	No class specified
	1	Minor loss of function
	2	Moderate loss of function
	3	Major loss of function
	4	Extreme loss of function
CC (Chronic conditions)	0	No chronic conditions
	1	Having one chronic condition
	2	Having two chronic conditions
	3	Having more than two chronic conditions

**Table 2:** Characteristics of patients among SEX. Chi-square test is used to calculate *p* values for differences across male (SEX = 0) and female (SEX = 1).

Variable	Count	%	Count		%		*p* Value
			SEX = 0	SEX = 1	SEX = 0	SEX = 1	
**Total**	893,967	100.00	387,778	506,189	43.38	56.62	
**AGE**							
0	44,515	4.98	13,472	31,043	30.26	69.74	<0.001
1	300,732	33.64	112,553	188,179	37.43	62.57	<0.001
2	153,446	17.16	82,526	70,920	53.78	46.22	<0.001
3	395,274	44.22	179,227	216,047	45.34	54.66	<0.001
**RACE**							
1	623,895	69.79	277,462	346,433	44.47	55.53	<0.001
2	120,984	13.53	50,687	70,297	41.90	58.10	<0.001
3	82,115	9.19	32,672	49,443	39.79	60.21	<0.001
4	22,311	2.50	7638	14,673	34.23	65.77	<0.001
5	1467	0.16	636	831	43.35	56.65	<0.001
6	43,195	4.83	18,683	24,512	43.25	56.75	<0.001
**CC**							
0	99,303	11.11	21,060	78,243	21.21	78.79	<0.001
1	95,063	10.63	37,038	58,025	38.96	61.04	<0.001
2	95,725	10.71	43,217	52,508	45.15	54.85	<0.001
3	603,876	67.55	286,463	317,413	47.44	52.56	<0.001

**Table 3:** Differences in characteristics of patients among CC. Chi-square test is used to calculate *p* values for differences across CC’s categories.

Variable	Count				%				*p* Value
	CC = 0	CC = 1	CC = 2	CC = 3	CC = 0	CC = 1	CC = 2	CC = 3	
**AGE**									
0	18,274	12,779	7233	6229	41.05	28.71	16.25	13.99	<0.01
1	73,362	58,543	46,806	122,021	24.39	19.47	15.56	40.57	<0.001
2	4204	11,641	17,788	119,813	2.74	7.59	11.59	78.08	<0.001
3	3463	12,100	23,898	355,813	0.88	3.06	6.05	90.02	<0.001
**SEX**									
0	21,060	37,038	43,217	286,463	5.43	9.55	11.14	73.87	<0.001
1	78,243	58,025	52,508	317,413	15.46	11.46	10.37	62.71	<0.001
**RACE**									
1	54,464	57,457	63,337	448,637	8.73	9.21	10.15	71.91	<0.001
2	13,801	15,450	14,852	76,881	11.41	12.77	12.28	63.55	<0.01
3	16,148	12,377	9950	43,640	19.67	15.07	12.12	53.14	<0.001
4	6615	3341	2326	10,029	29.65	14.97	10.43	44.95	<0.01
5	216	161	149	941	14.72	10.97	10.16	64.14	<0.001
6	8059	6277	5111	23,748	18.66	14.53	11.83	54.98	<0.001

**Table 4:** The disease severities and the differences in characteristics of patients. Chi-square test is used to calculate *p* values for differences across disease severity levels.

**ROM**	**Count**					**%**					***p* Value**
	**0**	**1**	**2**	**3**	**4**	**0**	**1**	**2**	**3**	**4**	
**Total**	275	435,408	223,616	171,232	63,436	0.03	48.71	25.01	19.15	7.10	
**AGE**											
0	24	39,224	3315	1261	691	0.05	88.11	7.45	2.83	1.55	<0.01
1	173	228,223	44,404	19,666	8266	0.06	75.89	14.77	6.54	2.75	<0.001
2	44	79,860	41,337	23,107	9098	0.03	52.04	26.94	15.06	5.93	<0.001
3	34	88,101	134,560	127,198	45,381	0.01	22.29	34.04	32.18	11.48	<0.001
**SEX**											
0	162	161,888	108,003	84,325	33,400	0.04	41.75	27.85	21.75	8.61	<0.001
1	113	273,520	115,613	86,907	30,036	0.02	54.04	22.84	17.17	5.93	<0.001
**RACE**											
1	184	283,403	162,417	129,563	48,328	0.03	45.42	26.03	20.77	7.75	<0.001
2	52	62,437	29,924	21,190	7381	0.04	51.61	24.73	17.51	6.10	<0.01
3	28	49,823	17,455	10,933	3876	0.03	60.67	21.26	13.31	4.72	<0.001
4	4	13,845	4089	3089	1284	0.02	62.05	18.33	13.85	5.76	<0.01
5	0	793	345	243	86	0.00	54.06	23.52	16.56	5.86	<0.001
6	7	25,107	9386	6214	2481	0.02	58.12	21.73	14.39	5.74	<0.001
CC											
0	128	93,166	3644	1433	932	0.13	93.82	3.67	1.44	0.94	<0.01
1	24	77,677	10,507	4397	2458	0.03	81.71	11.05	4.63	2.59	<0.001
2	25	65,597	17,322	8,590	4191	0.03	68.53	18.10	8.97	4.38	<0.001
3	98	198,968	192,143	156,812	55,855	0.02	32.95	31.82	25.97	9.25	<0.001
**SOI**	**Count**					**%**					***p* Value**
	**0**	**1**	**2**	**3**	**4**	**0**	**1**	**2**	**3**	**4**	
**Total**	275	234,553	359,761	231,742	67,636	0.03	26.24	40.24	25.92	7.57	
**AGE**											
0	24	21,003	17,846	4630	1012	0.05	47.18	40.09	10.40	2.27	<0.01
1	173	117,126	123,441	48,208	11,784	0.06	38.95	41.05	16.03	3.92	<0.001
2	44	36,981	62,747	41,168	12,506	0.03	24.10	40.89	26.83	8.15	<0.001
3	34	59,443	155,727	137,736	42,334	0.01	15.04	39.40	34.85	10.71	<0.001
**SEX**											
0	162	83,751	155,851	111,874	36,140	0.04	21.60	40.19	28.85	9.32	<0.001
1	113	150,802	203,910	119,868	31,496	0.02	29.79	40.28	23.68	6.22	<0.001
**RACE**											
1	184	155,643	249,492	168,514	50,062	0.03	24.95	39.99	27.01	8.02	<0.001
2	52	28,994	51,013	32,226	8699	0.04	23.97	42.17	26.64	7.19	<0.01
3	28	27,215	33,161	17,281	4430	0.03	33.14	40.38	21.04	5.39	<0.001
4	4	8220	8347	4322	1418	0.02	36.84	37.41	19.37	6.36	<0.01
5	0	394	613	368	92	0.00	26.86	41.79	25.09	6.27	<0.001
6	7	14,087	17,135	9031	2935	0.02	32.61	39.67	20.91	6.79	<0.001
**CC**											
0	128	59,589	32,227	6243	1116	0.13	60.01	32.45	6.29	1.12	<0.01
1	24	42,868	38,338	10,807	3026	0.03	45.09	40.33	11.37	3.18	<0.001
2	25	33,784	41,326	15,547	5043	0.03	35.29	43.17	16.24	5.27	<0.001
3	98	98,312	247,870	199,145	58,451	0.02	16.28	41.05	32.98	9.68	<0.001

**Table 5:** Results of multinomial logistic regression models.

**For ROM Model (The Reference Is at ROM = 0)**								
**Coefficient (β)**	**Estimate** **(Standard Error)**				**Odds Ratio** **(Exp(β))**			
	i=1	i=2	i=3	i=4	i=1	i=2	i=3	i=4
$\beta_{i}^{\left(0\right)}$	5.947 **	2.494 **	1.473 **	1.305 **				
	(0.023)	(0.023)	(0.023)	(0.023)				
$\beta_{i1}^{\left(age\right)}$	−0.377 **	−0.051 *	−0.020 *	−0.171 **	0.686	0.950	0.980	0.843
	(0.022)	(0.022)	(0.022)	(0.022)				
$\beta_{i2}^{\left(age\right)}$	−0.195 **	0.573 **	0.766 **	0.603 **	0.823	1.774	2.151	1.828
	(0.027)	(0.027)	(0.027)	(0.028)				
$\beta_{i3}^{\left(age\right)}$	−0.035 *	1.776 **	2.487 **	2.245 **	0.966	5.906	12.025	9.440
	(0.029)	(0.029)	(0.029)	(0.029)				
$\beta_{i}^{\left(sex\right)}$	1.221 **	0.914 **	0.850 **	0.714 **	3.391	2.494	2.340	2.042
	(0.013)	(0.013)	(0.013)	(0.013)				
$\beta_{i2}^{\left(race\right)}$	−0.179 **	0.073 **	0.107 **	0.010 *	0.836	1.076	1.113	1.010
	(0.016)	(0.016)	(0.016)	(0.016)				
$\beta_{i3}^{\left(race\right)}$	0.358 **	0.442 **	0.338 **	0.251 **	1.430	1.556	1.402	1.285
	(0.021)	(0.021)	(0.021)	(0.021)				
$\beta_{i4}^{\left(race\right)}$	1.030 **	1.031 **	1.047 **	1.115 **	2.801	2.804	2.849	3.050
	(0.051)	(0.051)	(0.051)	(0.051)				
$\beta_{i5}^{\left(race\right)}$	0.656 **	0.665 **	0.663 **	0.655 **	1.927	1.944	1.941	1.925
	(0.005)	(0.005)	(0.006)	(0.008)				
$\beta_{i6}^{\left(race\right)}$	1.004 **	1.027 **	0.932 **	0.967 **	2.729	2.793	2.540	2.630
	(0.038)	(0.038)	(0.038)	(0.038)				
$\beta_{i1}^{\left(cc\right)}$	1.782 **	2.769 **	2.679 **	2.533 **	5.942	15.943	14.571	12.591
	(0.023)	(0.023)	(0.023)	(0.023)				
$\beta_{i2}^{\left(cc\right)}$	1.667 **	3.119 **	3.094 **	2.825 **	5.296	22.624	22.065	16.861
	(0.023)	(0.023)	(0.023)	(0.023)				
$\beta_{i3}^{\left(cc\right)}$	1.311 **	3.687 **	4.005 **	3.442 **	3.710	39.925	54.872	31.249
	(0.016)	(0.016)	(0.016)	(0.016)				
**For SOI Model (The Reference Is at SOI = 0)**								
**Coefficient (α)**	**Estimate** **(Standard Error)**				**Odds Ratio** **(Exp(α))**			
	i=1	i=2	i=3	i=4	i=1	i=2	i=3	i=4
$\alpha_{i}^{\left(0\right)}$	5.489	4.980	3.289	1.618				
	(0.023)	(0.023)	(0.023)	(0.023)				
$\alpha_{i1}^{\left(age\right)}$	−0.389 **	−0.441 **	−0.319 **	−0.241 **	0.678	0.643	0.727	0.786
	(0.022)	(0.022)	(0.022)	(0.022)				
$\alpha_{i2}^{\left(age\right)}$	−0.150 **	−0.178 **	0.207 **	0.476 **	0.861	0.837	1.230	1.610
	(0.027)	(0.027)	(0.027)	(0.028)				
$\alpha_{i3}^{\left(age\right)}$	0.511 **	0.831 **	1.477 **	1.778 **	1.667	2.296	4.380	5.918
	(0.030)	(0.030)	(0.030)	(0.030)				
$\alpha_{i}^{\left(sex\right)}$	1.207 **	1.058 **	0.924 **	0.729 **	3.343	2.881	2.519	2.073
	(0.013)	(0.013)	(0.013)	(0.013)				
$\alpha_{i2}^{\left(race\right)}$	−0.278 **	−0.005 **	0.109 *	0.057 **	0.757	0.995	1.115	1.059
	(0.016)	(0.016)	(0.016)	(0.016)				
$\alpha_{i3}^{\left(race\right)}$	0.339 **	0.365 **	0.346 **	0.246 **	1.404	1.441	1.413	1.279
	(0.021)	(0.021)	(0.021)	(0.021)				
$\alpha_{i4}^{\left(race\right)}$	1.370 **	1.351 **	1.342 **	1.470 **	3.935	3.861	3.827	4.349
	(0.059)	(0.059)	(0.059)	(0.059)				
$\alpha_{i5}^{\left(race\right)}$	0.362 **	0.377 **	0.380 **	0.366 **	1.436	1.458	1.462	1.442
	(0.005)	(0.004)	(0.005)	(0.008)				
$\alpha_{i6}^{\left(race\right)}$	1.136 **	1.120 **	1.054 **	1.169 **	3.114	3.065	2.869	3.219
	(0.040)	(0.040)	(0.040)	(0.040)				
$\alpha_{i1}^{\left(cc\right)}$	1.591 **	2.034 **	2.297 **	2.660 **	4.909	7.645	9.944	14.296
	(0.023)	(0.023)	(0.023)	(0.023)				
$\alpha_{i2}^{\left(cc\right)}$	1.321 **	2.035 **	2.489 **	2.946 **	3.747	7.652	12.049	19.030
	(0.022)	(0.022)	(0.022)	(0.022)				
$\alpha_{i3}^{\left(cc\right)}$	0.855 **	2.189 **	3.230 **	3.516 **	2.351	8.926	25.280	33.650
	(0.016)	(0.016)	(0.016)	(0.016)				

** significant at 0.001, * significant at 0.05.

**Table 6:** Differences between the levels of SOI across the levels of ROM.

	ROM = 0	ROM = 1	ROM = 2	ROM = 3	ROM = 4
SOI = 0	100	0.00	0.00	0.00	0.00
SOI = 1	0.00	91.40	8.04	0.56	0.00
SOI = 2	0.00	55.28	37.35	7.30	0.07
SOI = 3	0.00	9.38	29.49	54.46	6.67
SOI = 4	0.00	0.63	3.00	25.81	70.56

## Data Availability

Data sharing not applicable.

## Appendix A

**Table A1:** Predicted probabilities corresponding to patient characteristics and ROM and SOI levels. The mean value (with its standard error) is reported, (calculation unit: %).

Characteristics of Patient	ROM Levels					SOI Levels				
	0	1	2	3	4	0	1	2	3	4
AGE = 0	0.04	83.43	10.28	4.10	2.15	0.03	40.78	42.98	13.13	3.08
	(0.06)	(11.67)	(6.82)	(3.49)	(1.44)	(0.06)	(13.76)	(5.65)	(6.99)	(1.77)
AGE = 1	0.05	79.10	13.08	5.34	2.43	0.05	41.15	41.08	14.14	3.59
	(0.08)	(13.84)	(8.13)	(4.31)	(1.51)	(0.08)	(14.02)	(5.26)	(7.43)	(2.04)
AGE = 2	0.04	70.95	17.17	8.12	3.71	0.04	38.62	38.89	17.18	5.27
	(0.07)	(17.06)	(9.36)	(5.87)	(2.00)	(0.06)	(14.19)	(3.98)	(8.39)	(2.82)
AGE = 3	0.02	45.60	25.89	19.74	8.74	0.02	29.50	40.71	22.63	7.14
	(0.04)	(20.08)	(8.64)	(9.38)	(2.66)	(0.03)	(12.70)	(2.58)	(9.24)	(3.35)
SEX = 0	0.06	66.85	17.89	10.22	4.98	0.05	35.34	41.21	17.86	5.53
	(0.08)	(22.31)	(10.26)	(9.08)	(3.57)	(0.08)	(14.03)	(4.31)	(8.97)	(3.26)
SEX = 1	0.02	72.69	15.33	8.43	3.54	0.02	39.68	40.62	15.68	4.01
	(0.03)	(20.62)	(9.89)	(8.32)	(2.84)	(0.03)	(14.46)	(5.11)	(8.57)	(2.52)
RACE = 1	0.18	88.41	5.95	3.30	2.16	0.16	56.60	33.46	8.07	1.71
	(0.09)	(21.58)	(9.91)	(8.78)	(3.40)	(0.08)	(14.60)	(4.75)	(8.78)	(3.00)
RACE = 2	0.07	66.50	18.17	10.68	4.58	0.07	32.60	42.93	19.19	5.22
	(0.10)	(22.74)	(10.47)	(9.53)	(3.36)	(0.09)	(13.53)	(4.01)	(9.36)	(3.11)
RACE = 3	0.04	70.28	17.01	8.86	3.81	0.04	38.71	40.72	16.29	4.24
	(0.06)	(21.65)	(10.63)	(8.50)	(3.02)	(0.06)	(14.47)	(4.81)	(8.81)	(2.73)
RACE = 4	0.02	70.48	15.68	9.20	4.61	0.01	39.28	39.53	15.96	5.22
	(0.03)	(21.71)	(9.77)	(8.79)	(3.64)	(0.02)	(14.71)	(4.71)	(8.58)	(3.34)
RACE = 5	0.00	69.65	16.72	9.50	4.13	0.00	35.85	42.30	17.55	4.30
	(0.00)	(21.95)	(10.26)	(8.96)	(3.21)	(0.00)	(14.04)	(4.44)	(9.10)	(2.68)
RACE = 6	0.02	70.96	16.28	8.58	4.16	0.02	39.63	40.06	15.34	4.95
	(0.03)	(21.47)	(10.30)	(8.32)	(3.33)	(0.03)	(14.65)	(4.89)	(8.37)	(3.20)
CC = 0	0.10	87.90	6.36	3.44	2.19	0.09	54.91	34.76	8.47	1.78
	(0.10)	(9.58)	(4.15)	(3.41)	(2.07)	(0.10)	(6.30)	(3.01)	(2.92)	(0.86)
CC = 1	0.02	76.26	13.64	6.49	3.59	0.01	42.07	41.16	12.89	3.86
	(0.02)	(15.35)	(7.01)	(5.55)	(2.89)	(0.02)	(6.46)	(2.24)	(3.79)	(1.68)
CC = 2	0.02	68.07	18.37	9.08	4.47	0.01	34.32	43.77	16.48	5.41
	(0.02)	(17.69)	(7.78)	(6.92)	(3.16)	(0.02)	(6.15)	(2.11)	(4.31)	(2.18)
CC = 3	0.01	46.83	28.06	18.30	6.80	0.01	18.75	43.97	29.24	8.02
	(0.02)	(18.43)	(6.05)	(9.68)	(3.20)	(0.01)	(4.49)	(3.94)	(5.44)	(2.68)
AGE = 0, CC = 0	0.10	95.35	2.83	0.97	0.75	0.09	58.04	34.73	6.09	1.04
	(0.09)	(0.97)	(0.50)	(0.21)	(0.20)	(0.09)	(3.71)	(2.53)	(0.99)	(0.24)
AGE = 0, CC = 1	0.02	89.20	7.08	2.21	1.49	0.01	45.58	42.39	9.65	2.37
	(0.01)	(1.98)	(1.16)	(0.46)	(0.38)	(0.01)	(3.83)	(2.19)	(1.35)	(0.52)
AGE = 0, CC = 2	0.02	83.79	10.57	3.52	2.10	0.01	37.84	46.03	12.68	3.43
	(0.02)	(2.78)	(1.61)	(0.70)	(0.52)	(0.01)	(3.70)	(1.76)	(1.58)	(0.71)
AGE = 0, CC = 3	0.02	65.36	20.65	9.69	4.28	0.01	21.64	48.77	24.09	5.48
	(0.02)	(4.65)	(2.35)	(1.54)	(0.91)	(0.01)	(2.81)	(0.86)	(2.16)	(1.01)
AGE = 1, CC = 0	0.14	93.75	3.85	1.36	0.91	0.13	58.70	33.34	6.60	1.22
	(0.13)	(1.28)	(0.66)	(0.30)	(0.24)	(0.14)	(3.75)	(2.43)	(1.07)	(0.29)
AGE = 1, CC = 1	0.02	85.77	9.42	3.04	1.75	0.02	46.08	40.67	10.45	2.78
	(0.02)	(2.49)	(1.47)	(0.61)	(0.44)	(0.02)	(3.90)	(2.09)	(1.45)	(0.60)
AGE = 1, CC = 2	0.02	79.03	13.79	4.74	2.42	0.02	38.19	44.07	13.71	4.01
	(0.02)	(3.37)	(1.96)	(0.89)	(0.58)	(0.02)	(3.77)	(1.66)	(1.69)	(0.82)
AGE = 1, CC = 3	0.02	57.86	25.26	12.22	4.64	0.02	21.63	46.23	25.79	6.34
	(0.02)	(4.98)	(2.49)	(1.75)	(0.93)	(0.02)	(2.86)	(0.90)	(2.25)	(1.15)
AGE = 2, CC = 0	0.11	90.17	5.75	2.39	1.58	0.10	56.62	32.90	8.48	1.90
	(0.11)	(1.90)	(0.95)	(0.50)	(0.41)	(0.10)	(3.89)	(2.24)	(1.33)	(0.44)
AGE = 2, CC = 1	0.02	78.61	13.40	5.08	2.90	0.02	43.44	39.22	13.11	4.22
	(0.02)	(3.46)	(1.89)	(0.95)	(0.69)	(0.02)	(3.98)	(1.77)	(1.72)	(0.89)
AGE = 2, CC = 2	0.02	69.67	18.86	7.61	3.84	0.01	35.37	41.75	16.90	5.97
	(0.01)	(4.33)	(2.31)	(1.28)	(0.85)	(0.01)	(3.79)	(1.33)	(1.93)	(1.18)
AGE = 2, CC = 3	0.01	45.35	30.68	17.42	6.54	0.01	19.07	41.69	30.24	8.99
	(0.01)	(5.09)	(2.24)	(2.03)	(1.15)	(0.01)	(2.71)	(1.11)	(2.33)	(1.54)
AGE = 3, CC = 0	0.07	72.35	13.02	9.04	5.52	0.04	46.28	38.04	12.70	2.93
	(0.07)	(4.31)	(1.64)	(1.54)	(1.23)	(0.04)	(4.00)	(1.89)	(1.74)	(0.63)
AGE = 3, CC = 1	0.01	51.47	24.66	15.63	8.23	0.01	33.19	42.38	18.35	6.08
	(0.01)	(5.25)	(2.06)	(1.98)	(1.51)	(0.01)	(3.69)	(1.21)	(2.02)	(1.18)
AGE = 3, CC = 2	0.01	39.81	30.25	20.43	9.51	0.01	25.88	43.22	22.65	8.25
	(0.01)	(5.02)	(1.85)	(2.10)	(1.55)	(0.01)	(3.27)	(0.99)	(2.13)	(1.50)
AGE = 3, CC = 3	0.00	18.76	35.65	33.87	11.72	0.00	12.67	39.20	36.84	11.28
	(0.00)	(3.21)	(1.11)	(1.96)	(1.47)	(0.00)	(2.00)	(1.55)	(2.25)	(1.80)
SEX = 0, CC = 0	0.16	86.12	7.12	3.94	2.66	0.14	52.62	35.84	9.28	2.11
	(0.12)	(10.59)	(4.46)	(3.81)	(2.39)	(0.12)	(6.04)	(2.69)	(3.02)	(0.92)
SEX = 0, CC = 1	0.02	73.47	14.97	7.27	4.27	0.02	39.69	41.82	13.93	4.54
	(0.02)	(16.31)	(7.19)	(6.00)	(3.22)	(0.02)	(6.05)	(2.04)	(3.84)	(1.76)
SEX = 0, CC = 2	0.02	64.79	19.91	10.03	5.24	0.02	32.02	44.01	17.64	6.31
	(0.02)	(18.27)	(7.69)	(7.30)	(3.44)	(0.02)	(5.65)	(2.23)	(4.31)	(2.27)
SEX = 0, CC = 3	0.02	43.02	29.54	19.65	7.77	0.02	17.05	43.18	30.59	9.16
	(0.02)	(17.70)	(5.30)	(9.69)	(3.25)	(0.02)	(3.95)	(4.21)	(5.25)	(2.70)
SEX = 1, CC = 0	0.05	89.69	5.60	2.94	1.72	0.04	57.20	33.67	7.65	1.44
	(0.04)	(8.28)	(3.74)	(2.95)	(1.62)	(0.04)	(5.82)	(2.96)	(2.62)	(0.64)
SEX = 1, CC = 1	0.01	79.06	12.31	5.71	2.91	0.01	44.45	40.50	11.85	3.18
	(0.01)	(14.13)	(6.72)	(5.08)	(2.39)	(0.01)	(6.08)	(2.27)	(3.52)	(1.30)
SEX = 1, CC = 2	0.01	71.36	16.82	8.12	3.69	0.01	36.62	43.52	15.33	4.52
	(0.01)	(16.84)	(7.72)	(6.52)	(2.70)	(0.01)	(5.86)	(2.01)	(4.08)	(1.72)
SEX = 1, CC = 3	0.01	50.64	26.59	16.94	5.82	0.01	20.45	44.77	27.89	6.88
	(0.01)	(18.73)	(6.50)	(9.68)	(2.89)	(0.01)	(4.41)	(3.56)	(5.39)	(2.16)
RACE = 1, CC = 0	0.06	70.74	15.79	9.13	4.28	0.06	39.00	39.96	16.29	4.68
	(0.11)	(9.70)	(4.11)	(3.46)	(2.17)	(0.11)	(5.90)	(2.52)	(2.83)	(0.86)
RACE = 1, CC = 1	0.03	77.21	12.88	6.30	3.58	0.03	43.75	40.04	12.42	3.77
	(0.02)	(15.84)	(7.10)	(5.74)	(3.07)	(0.02)	(6.23)	(1.68)	(3.74)	(1.71)
RACE = 1, CC = 2	0.03	69.21	17.42	8.87	4.48	0.03	35.87	42.82	15.98	5.30
	(0.02)	(18.44)	(7.95)	(7.22)	(3.39)	(0.02)	(6.03)	(1.65)	(4.29)	(2.23)
RACE = 1, CC = 3	0.03	48.11	26.91	18.07	6.88	0.02	19.79	43.53	28.71	7.95
	(0.02)	(19.63)	(6.29)	(10.26)	(3.47)	(0.02)	(4.52)	(3.95)	(5.48)	(2.74)
RACE = 2, CC = 0	0.20	85.87	7.30	4.16	2.47	0.18	49.32	38.18	10.26	2.06
	(0.13)	(11.38)	(4.81)	(4.22)	(2.40)	(0.13)	(6.04)	(2.17)	(3.36)	(0.99)
RACE = 2, CC = 1	0.03	73.12	15.27	7.63	3.94	0.03	36.60	43.86	15.16	4.35
	(0.02)	(17.33)	(7.67)	(6.57)	(3.18)	(0.02)	(5.92)	(1.57)	(4.19)	(1.87)
RACE = 2, CC = 2	0.03	64.39	20.25	10.50	4.83	0.03	29.24	45.73	19.02	5.98
	(0.02)	(19.35)	(8.16)	(7.96)	(3.37)	(0.02)	(5.46)	(2.22)	(4.65)	(2.38)
RACE = 2, CC = 3	0.03	42.61	29.85	20.43	7.09	0.02	15.24	43.94	32.32	8.48
	(0.02)	(18.71)	(5.60)	(10.46)	(3.18)	(0.02)	(3.72)	(4.72)	(5.53)	(2.76)
RACE = 3, CC = 0	0.12	88.25	6.46	3.23	1.94	0.12	56.19	34.08	8.06	1.55
	(0.08)	(9.74)	(4.46)	(3.38)	(1.94)	(0.08)	(5.86)	(2.56)	(2.83)	(0.78)
RACE = 3, CC = 1	0.02	76.75	13.91	6.13	3.19	0.02	43.41	40.77	12.40	3.40
	(0.01)	(15.87)	(7.64)	(5.57)	(2.73)	(0.01)	(6.15)	(1.71)	(3.75)	(1.55)
RACE = 3, CC = 2	0.02	68.62	18.78	8.60	3.98	0.02	35.59	43.62	15.97	4.80
	(0.01)	(18.41)	(8.53)	(6.99)	(3.01)	(0.01)	(5.93)	(1.63)	(4.32)	(2.03)
RACE = 3, CC = 3	0.02	47.49	28.90	17.48	6.11	0.02	19.66	44.39	28.73	7.20
	(0.01)	(19.40)	(6.75)	(9.95)	(3.09)	(0.01)	(4.44)	(3.89)	(5.57)	(2.51)
RACE = 4, CC = 0	0.06	88.31	5.93	3.35	2.34	0.04	57.03	33.11	7.91	1.91
	(0.04)	(9.89)	(4.09)	(3.49)	(2.34)	(0.03)	(5.93)	(2.50)	(2.77)	(0.96)
RACE = 4, CC = 1	0.01	76.95	12.81	6.36	3.87	0.01	44.05	39.59	12.16	4.20
	(0.01)	(16.00)	(7.00)	(5.77)	(3.30)	(0.00)	(6.30)	(1.67)	(3.65)	(1.90)
RACE = 4, CC = 2	0.01	68.90	17.31	8.94	4.83	0.01	36.11	42.33	15.64	5.91
	(0.01)	(18.57)	(7.82)	(7.23)	(3.63)	(0.00)	(6.11)	(1.67)	(4.17)	(2.48)
RACE = 4, CC = 3	0.01	47.76	26.66	18.16	7.41	0.01	19.94	43.07	28.12	8.87
	(0.01)	(19.62)	(6.12)	(10.21)	(3.69)	(0.00)	(4.59)	(3.99)	(5.30)	(3.04)
RACE = 5, CC = 0	0.00	87.94	6.42	3.51	2.13	0.00	53.02	36.37	8.98	1.62
	(0.00)	(10.13)	(4.39)	(3.65)	(2.12)	(0.00)	(5.94)	(2.43)	(3.06)	(0.80)
RACE = 5, CC = 1	0.00	76.15	13.75	6.62	3.48	0.00	40.21	42.71	13.57	3.51
	(0.00)	(16.23)	(7.41)	(5.95)	(2.94)	(0.00)	(6.01)	(1.60)	(3.97)	(1.56)
RACE = 5, CC = 2	0.00	67.92	18.50	9.25	4.33	0.00	32.61	45.21	17.29	4.89
	(0.00)	(18.69)	(8.19)	(7.41)	(3.21)	(0.00)	(5.68)	(1.80)	(4.50)	(2.02)
RACE = 5, CC = 3	0.00	46.59	28.22	18.62	6.57	0.00	17.57	44.90	30.37	7.17
	(0.00)	(19.38)	(6.26)	(10.32)	(3.22)	(0.00)	(4.08)	(4.23)	(5.65)	(2.44)
RACE = 6, CC = 0	0.06	88.64	6.11	3.09	2.09	0.05	57.29	33.34	7.53	1.79
	(0.04)	(9.56)	(4.25)	(3.24)	(2.10)	(0.04)	(5.85)	(2.58)	(2.66)	(0.91)
RACE = 6, CC = 1	0.01	77.38	13.24	5.90	3.47	0.01	44.40	40.01	11.62	3.96
	(0.01)	(15.67)	(7.35)	(5.39)	(2.99)	(0.01)	(6.23)	(1.73)	(3.53)	(1.80)
RACE = 6, CC = 2	0.01	69.40	17.93	8.31	4.35	0.01	36.50	42.90	15.00	5.59
	(0.01)	(18.29)	(8.28)	(6.81)	(3.31)	(0.01)	(6.05)	(1.62)	(4.07)	(2.37)
RACE = 6, CC = 3	0.01	48.43	27.83	17.02	6.72	0.01	20.32	44.01	27.20	8.46
	(0.01)	(19.57)	(6.69)	(9.81)	(3.44)	(0.01)	(4.59)	(3.88)	(5.27)	(2.94)
